# The Effect of a Virtual Reality Counseling Program Based on Metacognitive Therapy in Reducing Post-Traumatic Stress Disorder among Those Recovering from Covid-19

**DOI:** 10.1192/j.eurpsy.2024.214

**Published:** 2024-08-27

**Authors:** N. S. G. Abdelrasheed, M. M. M. Al Majali

**Affiliations:** Department of Education, Dhofar University, Salalah, Oman

## Abstract

**Introduction:**

Many COVID-19 survivors who were attacked and suffered severe symptoms of the virus have suffered from post-traumatic stress disorder (PTSD) which persists for long periods. These people need treatment to alleviate the severity of these disorders. Metacognitive therapy (MCT) is one of the modern therapeutic trends in psychological counseling, which focuses on the nature of the thought rather than on identifying and changing the thought as in other cognitive therapies. It is also concerned with whether people possess an aspect of reflective awareness and aims for a broader understanding of the way the mind works. Working on the process of metacognition, that is, the individual’s thinking about what he knows, being aware of his thoughts, and constantly monitoring and organizing them, helps reduce anxiety disorders and mood swings, and this will reduce psychotic disorders.

**Objectives:**

The current study aims to identify the effect of a virtual reality (VR) counseling program based on MCT in reducing the severity of PTSD among survivors of Covid-19. It also examines the continuity of the effectiveness of this program in reducing these disorders.

**Methods:**

The quasi-experimental method (two group design) with experimental and control groups with a pre-posttest and a follow-up test was adopted. The sample for the current study consisted of 60 COVID-19 survivors suffering from PTSD. The PTSD scale was applied online to a group of people recovering from Covid-19 from the Arab Republic of Egypt. Then those who had high scores were selected, contacted and their consent was obtained to apply a virtual reality counseling program to them. The counseling program was implemented via virtual reality technology, and consisted of 20 counseling sessions, each session lasted between 60-90 minutes. The program continued for two months, with two sessions per week.

**Results:**

The results of the current research revealed a significant improvement in the experimental group through a significant reduction in their post-traumatic stress disorders. The results also showed the effectiveness of the counseling program based on metacognitive therapy in reducing the manifestations of post-traumatic stress disorders in those recovering from Covid-19. The results confirmed the continuing effect of the program after the follow-up period.

**Conclusions:**

Using metacognitive therapy has an effective effect in reducing post-traumatic stress disorder, and it can be used with many psychologically disturbed people.

**Disclosure of Interest:**

None Declared

